# Functionalized carbon nanotubes mixed matrix membranes of polymers of intrinsic microporosity for gas separation

**DOI:** 10.1186/1556-276X-7-504

**Published:** 2012-09-06

**Authors:** Muntazim Munir Khan, Volkan Filiz, Gisela Bengtson, Sergey Shishatskiy, Mushfequr Rahman, Volker Abetz

**Affiliations:** 1 Institute of Polymer Research, Helmholtz-Zentrum Geesthacht, Max-Planck-StraSSe 1, 21502, Geesthacht, Germany

**Keywords:** Mixed matrix membrane, Multi-walled carbon nanotubes, Polymer of intrinsic microporosity

## Abstract

The present work reports on the gas transport behavior of mixed matrix membranes (MMM) which were prepared from multi-walled carbon nanotubes (MWCNTs) and dispersed within polymers of intrinsic microporosity (PIM-1) matrix. The MWCNTs were chemically functionalized with poly(ethylene glycol) (PEG) for a better dispersion in the polymer matrix. MMM-incorporating functionalized MWCNTs (f-MWCNTs) were fabricated by dip-coating method using microporous polyacrylonitrile membrane as a support and were characterized for gas separation performance. Gas permeation measurements show that MMM incorporated with pristine or functionalized MWCNTs exhibited improved gas separation performance compared to pure PIM-1. The f-MWCNTs MMM show better performance in terms of permeance and selectivity in comparison to pristine MWCNTs. The gas permeances of the derived MMM are increased to approximately 50% without sacrificing the selectivity at 2 wt.% of f-MWCNTs' loading. The PEG groups on the MWCNTs have strong interaction with CO_2_ which increases the solubility of polar gas and limit the solubility of nonpolar gas, which is advantageous for CO_2_/N_2_ selectivity. The addition of f-MWCNTs inside the polymer matrix also improved the long-term gas transport stability of MMM in comparison with PIM-1. The high permeance, selectivity, and long term stability of the fabricated MMM suggest that the reported approach can be utilized in practical gas separation technology.

## Background

During the last two decades, significant improvements in the performance of polymeric materials for gas separation membranes have been made [1-6], and understanding of the relationships between the polymer structure and gas transport properties of polymeric membranes has been greatly advanced [2,3]. Despite these advantages and progresses, polymeric membranes are restricted by the trade-off trend between gas permeability and selectivity, as shown by Robeson [7]. Most of the researchers have paid special attention to the relationship between polymer structure and gas separation properties in order to improve membrane performance both in permeance and selectivity.

The permeability of a polymeric membrane is mainly controlled by the chain mobility, the packing density, and the free volume of the polymer structure. The introduction of rigid fillers having particle size close to the characteristic size of the macromolecules which form the selective polymer film can be the best technique to improve gas permeability by inhibiting molecular chain packing and increasing free volume. Therefore, mixed matrix membranes (MMM) defined as the synergistic combination of organic polymers with inorganic nanofillers (both permeable and impermeable) dispersed at the nanometer level have been studied as an alternative approach to solve the trade-off problem of polymeric membranes in gas separation [8,9].

Proper material selection for both the polymer matrix and the inorganic phase is important in the development of MMM. It has been found that polymer properties as well as inorganic phase properties affect mixed matrix membranes morphology and thus influence the separation performance [10]. Compared to the pure polymer membranes, many polymer-inorganic MMM show higher permeabilities without sacrificing or even improve gas selectivity. Pinnau and He reported an unexpected increase of gas permeability without loss of gas selectivity in a series of high-free-volume glassy polymers whereby inorganic nonporous nanoparticles, such as fumed silica or carbon black, were incorporated into the polymeric matrix [11]. Some experimental studies used carbon nanotubes as inorganic nanofillers to fabricate MMM, and an improvement of the gas permeability compared to the neat polymer membranes was observed. For instance, Kim et al. [12] reported on the addition of carbon nanotubes (CNTs) to poly(imide siloxane) membranes which resulted in increased O_2_, N_2_, and CH_4_ permeability. Cong et al. [13] prepared the brominated poly(2, 6-diphenyl-1,4-phenylene oxide) composite membrane with single-walled CNTs or MWCNTs and found that the low concentration of CNTs addition increases the gas permeability without sacrificing the selectivity. Weng et al. [14] fabricated the MWCNTs/PBNPI membrane. In their results both the permeabilities and the selectivities of H_2_, CO_2_, and CH_4_ improved significantly at high MWCNTs concentrations (>5 wt.%). Based on these investigations, one can conclude that the interaction between polymer matrix and nanotubes may disrupt the polymer chain packing thus enhancing gas diffusion due to introducing more free volume voids between the polymer chains and nanoscale defects on the polymer/nanofillers interface.

Tailoring the free volume cavities by controlling the macromolecule's size and shape of the microporous polymer directly influences gas transport properties [15]. In particular, a novel class of high free volume, glassy, ladder-type polymers, referred to as polymer of intrinsic microporosity (PIM), is a potential candidate for highly effective gas separation membranes comprising the capability for gas permeability and selectivity optimization by changing the polymer chain packing [16]. McKeown et al. [17,18] were the first to report this new class of rigid ladder-type polydioxanes containing highly contorted chains. Among these, PIM-1, containing the contorted spirobisindane unit, has attracted the most attention due to its relative ease of synthesizing high molecular weight polymers and the combining outstanding permeability with relatively moderate but technically attractive selectivity [19,20], especially for O_2_/N_2_ and CO_2_/CH_4_ pairs, which shows the upper bound trade-off introduced by Robeson [7]. However, a major drawback in the practical use of PIM-1 is the significant decay of its gas permeability with time.

In the present work, MMM were fabricated by loading of functionalized MWCNTs (f-MWCNTs) as an inorganic dispersed phase and PIM-1 as a polymer matrix. The MWCNTs were chosen because they had been proven to be promising nanofillers in tailoring polymeric material suited to be prescribed for application even at low incorporation [21]. To the best of our knowledge, so far there is no literature available on using MWCNTs combined with PIM-1 as polymer matrix for gas separation. However, it is well documented that for sufficient enhancement of MMM performance, the dispersion of MWCNTs in the polymer matrix should be very fine, which means that the surface interaction between the filler and the polymer matrices should be strong [10]. In response to that, the MWCNTs were functionalized with poly(ethylene glycol) (PEG) as a spacer via ‘grafting to’ method [22] to facilitate their dispersion in the PIM-1 matrix. The as-prepared MMM were characterized for their morphology using scanning electron microscopy (SEM). Gas permeability, permselectivity, and long term membrane stability were studied by pure gas permeation measurements.

## Methods

### Materials

The monomer 5,5^′^,6,6^′^-tetrahydroxy-3,3,3^′^,3^′^-tetramethyl-1,1^′^-spirobisindane (TTSBI, 97%) was obtained from ABCR (GmbH & Co. KG, Karlsruhe, Germany) and 2,3,5,6-tetrafluoroterephthalonitrile (TFTPN, 99%) was kindly donated by Lanxess (Bitterfeld, Germany). TFTPN was sublimated twice under vacuum prior to use. Potassium carbonate (K_2_CO_3_, >99.5%) was dried overnight under vacuum at 120°C in order to ensure no moisture is trapped in it and then milled in a ball mill for 15 min. The MWCNTs were supplied by FutureCarbon GmbH (Bayreuth, Germany) (purity >98%, surface area of 250 m^2^/g, diameter varies from 12 to 15 nm, and number of walls was 8 to 12). Poly(ethylene glycol) of 200 g/mol and diethylbenzene (isomeric mixture) was purchased from Sigma-Aldrich (Sigma-Aldrich Logistik GmbH, Schnelldorf, Germany); dimethyl acetamide (DMAc, ≥99%), nitric acid (HNO_3_, 65% *v/v*), thionyl chloride (SOCl_2_, ≥99%), tetrahydrofurane (THF, ≥ 99,9%), methanol (≥99.9%), chloroform (CHCl_3_, 99.99%), and dioxane (≥99%) from Merck (Merck, KGaA, Darmstadt, Germany) were used as received.

### Characterization

**Figure 1:**
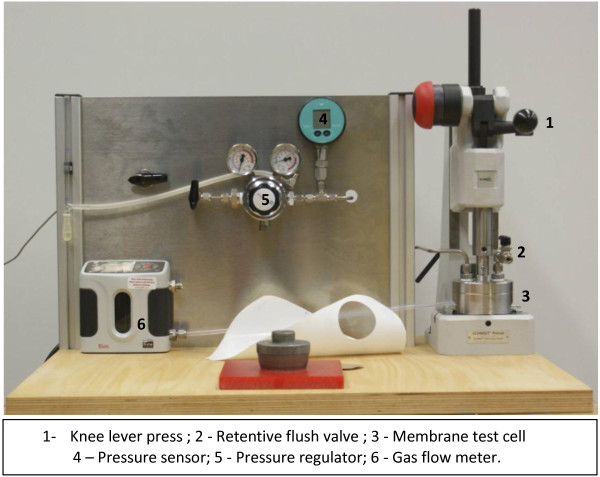
Gas permeation test facility.

Fourier transform infrared (FTIR) spectroscopy was conducted using a Bruker Equinox 55 (Bruker Optics, Bremen, Germany). The samples were mixed with KBr, and pellets were prepared under hydraulic press force of 10 t. Pellets were vacuum-dried at 35°C for 12 h. The transmission measurements were done in a spectral range of 400 to 4,000 cm^−1^ with a resolution of 4 cm^−1^ and average of 64 scans. Thermal gravimetric analysis (TGA) was used to investigate the weight changes of f-MWCNTs samples as a function of temperature using Netzsch TG209 F1 Iris instrument (NETZSCH-Gerätebau GmbH, Selb, Germany). The experiments were conducted under argon flow from 25°C to 900°C at 10 K/min. The weight loss was estimated from 100°C to 600°C in this study. A LEO Gemini 1550 VP instrument (Carl Zeiss AG, Oberkochen, Germany) equipped with field emission cathode operated at 1–1.5 kV was used to study the morphology of pure PIM-1 and MMM. SEM was also used to observe the compatibility between CNTs and the polymer matrix. For cross section analysis, the samples were fractured in liquid nitrogen in order to have distinct view of the membrane's selective layer section. Before scanning, the membrane samples were coated with Au/Pd in a sputter-coater. The permeation test involved the use of a gas permeation cell in which the membrane was placed on a sintered metal plate and pressurized at the feed side. Gas permeation rates were determined by a constant pressure variable volume system using a BIOS Definer™ 220 flow meter (Bios International Corporation, Butler, NJ, USA). Figure 1 illustrates the gas permeation test facility.

The pressure-normalized gas permeation flux or permeance for gas *i*, can be calculated as follows:

$$\;{\left(\frac{P}{l}\right)}_{i}=\frac{Q_{i}}{A\Delta p}$$

Where *Q*_*i*_ is the volumetric flow rate of gas *i* (normal cubic meter per hour), Δ*p* is the pressure difference across the membrane (bar), *A* is the membrane-effective surface area (square meters) and *l* is the membrane-separating layer thickness (centimeters). The ideal separation factor *α*_*i*/*j*_ was calculated by using the following equation:

$$\;\alpha_{i/j}=\frac{P_{i}}{P_{j}}$$

The long-term stability test was carried out at a similar facility at constant pressure (2 bar) and 27°C. The gas flow rate was measured manually using a flow meter (Bioblock Scientific, Cedex, France). All data presented in this study were collected for at least three stamps of MMM of the same origin in order to obtain statistically valid results.

### Experimental details

#### Functionalization of MWCNTs

##### (a) Oxidation of the MWCNTs

The MWCNTs were dispersed in HNO_3_ in a round bottom flask. After 30 min of sonication at room temperature, the solution was heated to 65°C and continuously stirred for 48 h. The dispersion was then filtered and washed with ample amounts of distilled water until the pH = 7 was achieved. Finally, the oxidized carbon nanotubes were dried under vacuum at 60°C for 72 h.

##### (b) Conversion of carboxylated MWCNTs to acyl chloride MWCNTs and esterification

The carboxyl groups of the oxidized MWCNTs were transformed into acid chloride groups by stirring the dispersion with SOCl_2_ for 24 h at 65°C. After reaction, the dispersion was generously washed with THF, filtered, and dried overnight at ambient temperature. Then acyl chloride-functionalized MWCNTs were added in PEG (200 g/mol) heated to 120°C and stirred for 48 h. Finally, functionalized carbon nanotubes were washed with THF and filtered through a Teflon® membrane (Sartorius Stedium Biotech GmbH, Göttingen, Germany) (0.2 μm pore size), followed by drying at ambient temperature for 48 h [22].

#### Synthesis and characterization of polymer of intrinsic microporosity (PIM-1)

PIM-1 was prepared by using a fast synthesis method originally developed by Du et al. [23]. A slightly modified procedure for PIM-1 [24] was carried out as follows: equimolar ratio of TTSBI and TFTPN was dissolved in DMAc to form an orange-red solution. The addition of K_2_CO_3_ (2.3 times with respect to -OH monomer concentration) caused the color change to yellow. The reaction mixture was immersed in an oil bath maintained at 150°C and kept under a continuous flow of argon. After 5 min the precipitate appeared and the reaction mixture became viscous, then diethylbenzene (DEB; same amount with respect to DMAc amount) was added into the reaction mixture, otherwise stirring could not be done easily. The stirring continued for a further 1 h, and the polymer was isolated by precipitation in methanol and filtered off. The polymer was boiled in water for several hours to remove salts and solvent residues, and then filtered off and dried overnight at 60°C. The dried polymer was dissolved in chloroform and reprecipitated again in methanol and then dried overnight at 60°C in vacuum to yield 90% PIM-1. The average molecular weight (*M*_w_) and polydispersity (*M*_w_/*M*_n_) of the prepared PIM-1 is 2.21 × 10^5^ (gmol^−1^) and 4.8, respectively, which are determined by size exclusion chromatography in chloroform against polystyrene standards.

#### Mixed matrix membrane preparation

PIM-1 was first dissolved in chloroform and stirred for 2 h. Chloroform dispersions of f-MWCNTs were homogenized for 40 min with an ultrasonic probe (Bandelin SONOPULS, Bandelin Electronic GmbH & Co. KG, Berlin, Germany) (frequency = 20 kHz) and blended with PIM-1 polymer solution and stirred overnight. The blend ratio of f-MWCNTs was 0.5, 1, 2, and 3 wt.% (in respect to polymer) with 1 wt.% polymer concentration in chloroform. A HZG in-house manufactured polyacrylonitrile (PAN) microporous membrane with an average pore size of 25 nm and with 15% surface porosity was used as a support for thin film composite membrane formation. The nitrogen permeance for PAN microporous membrane was around 120 Nm^3^/m^2^.h.bar at ambient temperature and shows the Knudsen selectivity. PIM-1/f-MWCNTs MMM were prepared by dip-coating using an in-house made lab dip coater (band having loop form of 10 to 20 cm wide and 100 cm long). The details of membrane fabrication conditions are shown in Table 1.

**Table 1:** Process parameters and membrane fabrication conditions

**Parameter**	**Condition**
Casting solution composition	1 wt.% PIM-1; 99 wt.% CHCl_3_; 0.5, 1, 2, and 3 wt.% CNT with respect to polymer
Casting temperature	Ambient temperature, 28°C
Casting speed	0.39 m/min
Drying procedure	Air dried (ambient conditions for 24 h)

## Results and discussion

### Functionalization of MWCNTs

**Figure 2:**
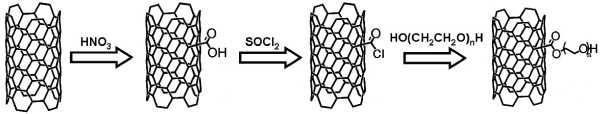
Functionalization of pristine MWCNTs via ‘grafting to’ method.

The surface modification of MWCNTs followed by covalent bonding of polymer chains on the surface of MWCNTs is depicted in Figure 2.

A similar procedure has been reported previously by other groups [25,26]. The first step shows the oxidation of pristine MWCNTs with HNO_3_. The aims of the chosen acid treatment are disaggregation of nanotube bundles, dissolution of the catalysts, and the removal of by-products (e.g amorphous carbon). Further reaction between the oxidized carbon nanotubes and thionyl chloride leads to the formation of acyl chloride groups. The acyl chloride groups are highly reactive and can further react with poly(ethylene glycol) to form esters. The pristine and functionalized MWCNTs were characterized by TGA and FTIR .

### Thermal gravimetric analysis

**Figure 3:**
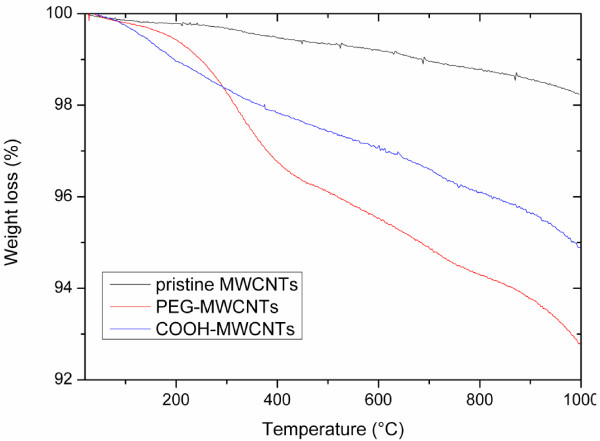
TGA analysis of pristine MWCNTs, carboxyl MWCNTs, and PEG-grafted MWCNTs.

The TGA of all three different samples of MWCNTs shows that no weight loss occurred below 100°C, which indicates the absence of a residual solvent. As revealed by the TGA curve shown in Figure 3, the pristine MWCNTs do not show significant weight loss up to 1,000°C. Both oxidized and PEG-grafted MWCNTs show weight loss between 100°C and 800°C. The weight loss observed after oxidation was 2 wt.% and after PEG grafting it was 4 wt.%. Hence, it can be concluded that about 2 wt.% of PEG were grafted on MWCNTs [27].

Although the reaction has been performed at high concentration of very low molecular weight PEG in the absence of the solvent, it is expected that during thorough washing processes, the entire unreacted PEG was washed out (washing was continued until constant mass loss in TGA was observed). Thus, the mass loss occurred only from the covalently attached PEG on the surface of the MWCNTs. Based on these results, the amount of PEG chains grafted on the MWCNTs was estimated (as above), and the concentration of PEG chains on the MWCNTs were calculated to be about 0.09 mmol/g.

### Fourier transform infrared spectroscopy

**Figure 4:**
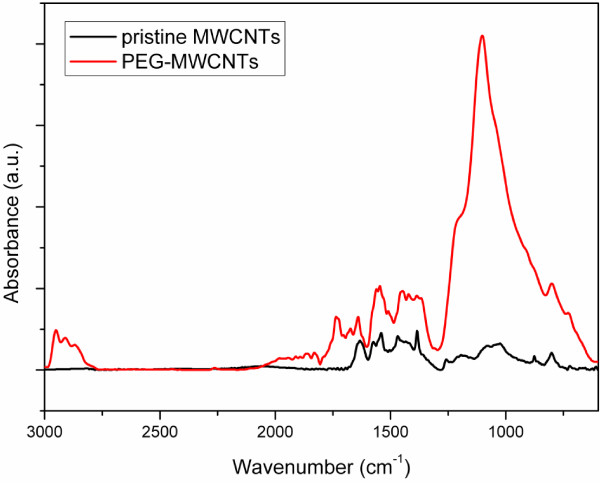
Infrared spectra of pristine MWCNTs and PEG-grafted MWCNTs.

Figure 4 shows the infrared spectrum of the pristine MWCNTs and PEG-grafted MWCNTs. The signals between 1,650 and 1,540 cm^−1^ indicated the C = C stretching mode of the aromatic ring. The peak at 1,724 cm^−1^ was attributed to the C = O stretching vibration of the ester carbonyl group. The stretching vibrations of the repeating -OCH_2_CH_2_ units of PEG and -COO bonds were observed at 1,092 and 1,240 cm^−1^, respectively, which is the evidence of a successful reaction. An asymmetric and symmetric stretching of C-H deformation is observed for PEG-MWCNTs at 2,927 and 2,853 cm^−1^ respectively, which is not observed in MWCNTs spectra but clearly observed in PEG-MWCNTs.

### Polymer (PIM-1) synthesis

**Figure 5:**
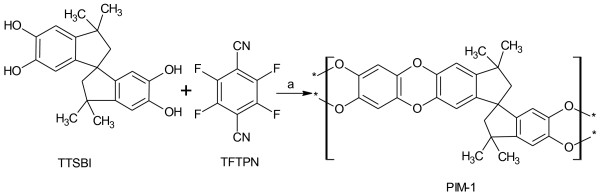
**Synthesis of PIM-1, a-reagent and condition, K**_**2**_**CO**_**3**_**, DMAc, and DEB at 150°C for 1 h.**

PIM-1 was synthesized by reacting with a molar equivalent of 5,5^′^,6,6^′^-tetrahydroxy-3,3,3^′^,3^′^-tetramethyl-1,1^′^-spirobisindane and 2,3,5,6-tetrafluoroterephthalonitrile with an excess of K_2_CO_3_ (Figure 5).

The polymer was synthesized based on the modified rapid synthesis of PIM-1. DEB was introduced to this reaction system instead of toluene, which is different from previous polymerization protocols [17,23], and seemed advantageous because of its higher boiling point (150°C) [28]. DEB was also added in the required minimal amounts to keep the mixture stirrable. The polymer was characterized by ^1^H nuclear magnetic resonance and IR spectroscopy.

### MMM morphology

#### Effect of pristine and f-MWCNTs on MMM morphology

**Figure 6:**
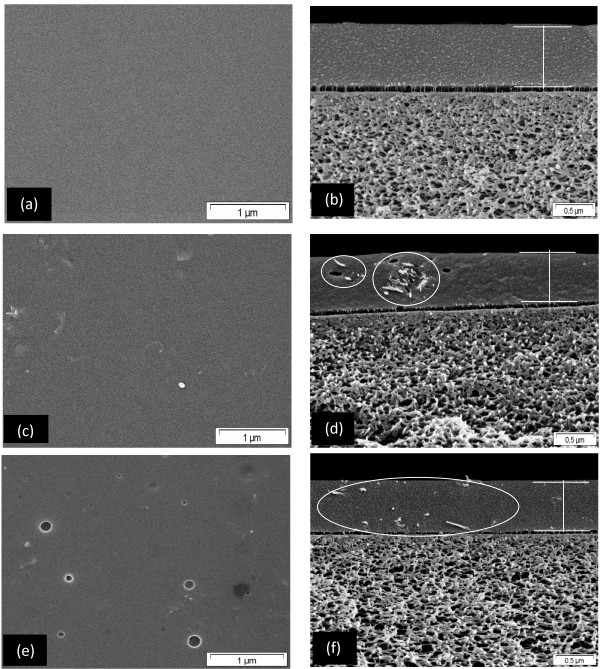
**SEM images of surface and cross section of PIM-1. (a, b)**, MMM of MWCNTs **(c, d)** and f-MWCNTs **(e, f).**

Figure 6 shows the SEM images of PIM-1 and PIM-1 incorporated with pristine and f- MWCNTs (1 wt.%) MMM. It was observed that the average thickness of all these membranes was 0.75 μm (Figure 6). When pristine MWCNTs were incorporated into the PIM-1 matrix, the resulting MMM contained agglomerated MWCNTs showed by white circle, which are clearly observable from the cross section (Figure 6d). The surface and cross section images of f-MWCNTs MMM indicated that most of the functionalized MWCNTs were well dispersed in PIM-1 matrix (Figure 6e, f). There was also no evidence of interfacial voids in the prepared MMM. The explanation for the agglomeration of MWCNTs in the polymer matrix was that the interactions between the MWCNTs (π-π interaction) are stronger than that with the polymer matrix [29-32]. Therefore, the pristine MWCNTs tend to agglomerate and do not distribute well in PIM-1 matrix. The presence of PEG on the surface of MWCNTs appeared to de-bundle the highly entangled MWCNTs which resulted in improved dispersion throughout the PIM-1 matrix.

### Effect of f-MWCNTs content on MMM morphology

**Figure 7:**
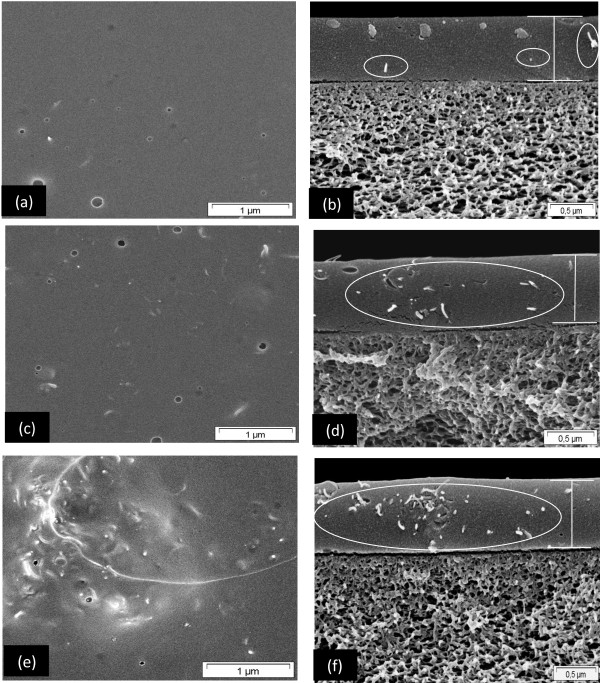
**SEM images of surface and cross section of PIM-1 MMM incorporated with different f-MWCNT loading.** 0.5 wt.% (**a, b**); 2 wt.% (**c, d**); and 3 wt.% (**e, f**) (PIM-1 and 1 wt.% f-MWCNT/PIM-1 MMM are shown in Figure 6a, b, e, f).

Figure 7 shows the surface and cross section morphology of PIM-1/f-MWCNTs MMM at different f-MWCNTs loading. Figure 6a shows the smooth surface of the pure PIM-1 membrane which was nearly defect-free while Figures 6e, f and 7a, b, c, d depicts the f-MWCNTs which tend to be well distributed throughout the polymer matrix independent of the f-MWCNTs loading (0.5 to 2 wt.%). No evidence of f-MWCNTs agglomeration or interface void was found even at higher magnification. As the MWCNTs loadings were further increased from 2.0 to 3.0 wt.%, the nanotubes tend to agglomerate and be not well distributed throughout the PIM-1 matrix. Also, the defects and interface voids around the f-MWCNTs agglomerates could be found on the surface of this MMM (Figure 7e, f). Therefore, more interface voids and agglomeration decreased the permeability of gases as the f-MWCNTs loading in PIM-1 matrix were increased from 2 to 3 wt.%.

From these observations, the threshold limit for the addition of CNTs to the polymer matrix to prevent agglomeration is typically around 3 wt.%, and the optimum limit for the addition of f-MWCNTs is 2 wt.% which corresponds to the significant improvement in the separation properties of the MMM, as discussed in ‘Effects of f-MWCNTs content on MMM gas separation performance’ Section.

### Gas permeation measurement

#### Effects of pristine and f-MWCNTs on MMM gas separation performance

The gas permeation properties of MMM were investigated by incorporating 1.0 wt.% of pristine and PEG-functionalized MWCNT in PIM-1 matrix. Permeance of CO_2_, CH_4_, N_2_, and O_2_ for pure PIM-1 and MMM as well as the respective permeability calculated from the thickness of selective layer are shown in Table 2.

**Table 2:** Comparison of gas permeation results of PIM-1, MMM of f-MWCNTs and MWCNTs

**Membrane**	**Permeance (Nm**^**3**^**/(m**^**2**^**h bar))**				**Permeability**^**a**^**(Barrer)**^**b**^			
	**O**_**2**_	**N**_**2**_	**CO**_**2**_	**CH**_**4**_	**O**_**2**_	**N**_**2**_	**CO**_**2**_	**CH**_**4**_
PIM-1	1.93	0.69	19.4	1.26	533	190	5,360	348
f-MWCNTs/PIM-1 MMM	2.19	0.78	25.7	2.47	605	215	7,090	682
Pristine MWCNTs/PIM-1 MMM	3.46	1.36	22.2	2.74	955	375	6,130	756

^a^Selective thin layer thickness considered constant as 0.75 μm from SEM images.

^b^$1Barrer=1\times 10^{-10}\cdot \frac{c{m^{3}}_{\left(STP\right)}\cdot cm}{cm^{2}\cdot \sec\cdot cmHg}$.

The permeation measurements were carried out using pure O_2_, N_2,_ CO_2_, and CH_4_ at 2 bar feed pressure at room temperature (27°C). It was observed that the MMM incorporated with pristine and PEG-functionalized MWCNTs have higher gas permeabilities compared to pure PIM-1. Moreover, the permeability of CO_2_ was higher for f-MWCNTs-incorporated MMM than for pristine MWCNTs-incorporated MMM. Table 3 shows the selectivity of CO_2_/N_2_, CO_2_/CH_4_, and O_2_/N_2_ gas pairs of PIM-1 and MMM prepared by incorporation of both pristine MWCNTs and f-MWCNTs.

**Table 3:** Comparison of various gas pair selectivities of PIM-1 and MMM of f-MWCNTs and MWCNTs

**Membrane**		**Selectivity**	
	**O**_**2**_**/N**_**2**_	**CO**_**2**_**/N**_**2**_	**CO**_**2**_**/CH**_**4**_
PIM-1	2.8	28.2	15.4
f-MWCNTs/PIM-1 MMM	2.8	32.9	10.4
Pristine MWCNTs/PIM-1 MMM	2.5	16.3	8.1

The CO_2_/CH_4_ selectivities have decreased in case of both f-MWCNTs and pristine MWCNTs-incorporated MMM. However, the observed CO_2_/CH_4_ selectivities are higher than that of MMM reported in literature using carboxyl-functionalized MWCNTs as inorganic filler in different glassy polymers (e.g., polyethersulfone and polyimide) [13,33].

From the results presented in Tables 4 and 5, it should be emphasized that the acid treatment of MWCNTs and consequent surface functionalization with PEG influenced both the permeability and selectivity of MMM. The most prevailing theory is that, after removing the catalyst particles, the amorphous carbon and the metal nanoparticles through the oxidation process with acidic mixtures, the surface area and micropore volume of CNTs increase and result in the occurence of adsorption sites. Few researchers [34,35] have investigated the physical adsorption of gas in CNTs and they found that the inner hollow cavities of CNTs could hold molecules or atoms by adsorption or capillarity, while Yang et al. [36] reported that the aggregated pores of high purity MWCNTs can adsorb N_2_ gas as extremely high as 750 mg/g.

**Table 4:** Gas permeation results of PIM-1, PIM-1 f-MWCNTs incorporated MMM

**Membrane**	**Permeance(Nm**^**3**^**/(m**^**2**^**h bar))**				**Permeability**^**a**^**(Barrer)**^**b**^			
	**O**_**2**_	**N**_**2**_	**CO**_**2**_	**CH**_**4**_	**O**_**2**_	**N**_**2**_	**CO**_**2**_	**CH**_**4**_
PIM1-1	1.93	0.69	19.4	1.26	533	190	5,360	348
0.5 wt.% f-MWCNTs/PIM-1	2.07				571	204	6,830	604
MMM								
1 wt.% f-MWCNTs/PIM-1	2.19	0.78	25.7	2.47	605	215	7,090	682
MMM								
2 wt.% f-MWCNTs/PIM-1	3.49	0.89	29.8	3.69	964	245	8,230	1,020
MMM								
3 wt.% f-MWCNTs/PIM-1	2.66	0.79	29.9	3.20	734	218	8,250	883
MMM								

^a^selective thin layer thickness considered at 0.75 μm from SEM images.

^b^$1Barrer=1\times 10^{-10}\cdot \frac{c{m^{3}}_{\left(STP\right)}\cdot cm}{cm^{2}\cdot \sec\cdot cmHg}$

**Table 5:** Various gas pair selectivity of PIM-1 and PIM-1 MMM-incorporated f-MWCNTs

**Membrane**		**Selectivity**	
	**O**_**2**_**/N**_**2**_	**CO**_**2**_**/N**_**2**_	**CO**_**2**_**/CH**_**4**_
PIM-1	2.80	28.2	15.4
0.5 wt.% f-MWCNTs/PIM-1	2.79	33.5	11.3
MMM			
1 wt.% f-MWCNTs/PIM-1	2.81	32.9	10.4
MMM			
2 wt.% f-MWCNTs/PIM-1	3.93	33.5	8.08
MMM			
3 wt.% f-MWCNTs/PIM-1	3.37	37.8	9.32

This could verify that the addition of pristine MWCNTs did not affect the selectivity of O_2_/N_2_. Interestingly, when PEG functionalization was applied onto MWCNTs, the selectivity of CO_2_/N_2_ of this MMM increased even at 1.0 wt.% loading of MWCNTs. This increase could be due to the higher compatibility of the polymer matrix with the surface of PEG-functionalized MWCNTs than that of pristine MWCNTs and preferential sorption of CO_2_ on the PEG chains of MWCNTs.

### Effects of f-MWCNTs content on MMM gas separation performance

In order to systematically investigate the effect of f-MWCNTs incorporation on the MMM gas separation performance, MMM were fabricated with different loadings of f-MWCNTs. The permeance and respective permeability (calculated from the thickness of selective layer using membrane's cross section SEM image) of O_2_, N_2_, CO_2_, and CH_4_ at different f-MWCNTs loadings are shown in Table 4. The order of gas permeance is CO_2_ > O_2_ > CH_4_ > N_2_. Gas permeance increased with f-MWCNTs loading from 0.5 to 2 wt.%. An increase in the free volume of the PIM-1 as a result of the disruption of the polymer chains packing due to the interaction between surface of PEG-functionalized MWCNTs and the PIM-1 chains might have contributed to the increment of the permeability. However, with 3 wt.% loading permeability of O_2_, N_2_, and CH_4_ decreased, except CO_2_, it was unchanged compared to 2 wt.% f-MWCNTs-incorporated MMM.

Permeation measurements used pure gas O_2_, N_2,_ CO_2_, CH_4_ of 2 bar feed pressure at room temperature (27°C). Table 5 shows the selectivity of O_2_/N_2_, CO_2_/N_2_, and CO_2_/CH_4_ for the PIM-1 and f-MWCNTs/PIM-1 MMM, measured at 2 bar. At 0.5 to 2 wt.% f-MWCNTs loading the selectivity of CO_2_/N_2_ and O_2_/N_2_ was found to be higher than for pure PIM-1. The high selectivity values of O_2_/N_2_ at 0.5 to 2 wt.% f-MWCNTs loading might have resulted due to the intimate interface interaction of f-MWCNTs-polymer chain. In contrary, the CO_2_/CH_4_ selectivity of the f-MWCNTs MMM is worse compared with the pure PIM-1 membrane. The functional groups on the MWCNTs have a significant role in gas selectivity. The PEG group, confirmed by FTIR in Figure 4, has stronger interaction with a polar gas, such as CO_2_ than a nonpolar gas, e.g., N_2_. In that case the polar gas solubility can be enhanced and the gas permeability be increased which facilitates the improvement of the total CO_2_/N_2_ selectivity. However, there is no improvement in the CO_2_/CH_4_ selectivity as f-MWCNTs loading increased from 0.5 to 3 wt.% in the polymer matrix.

### Effect of f-MWCNTs on long-term stability of MMM

**Figure 8:**
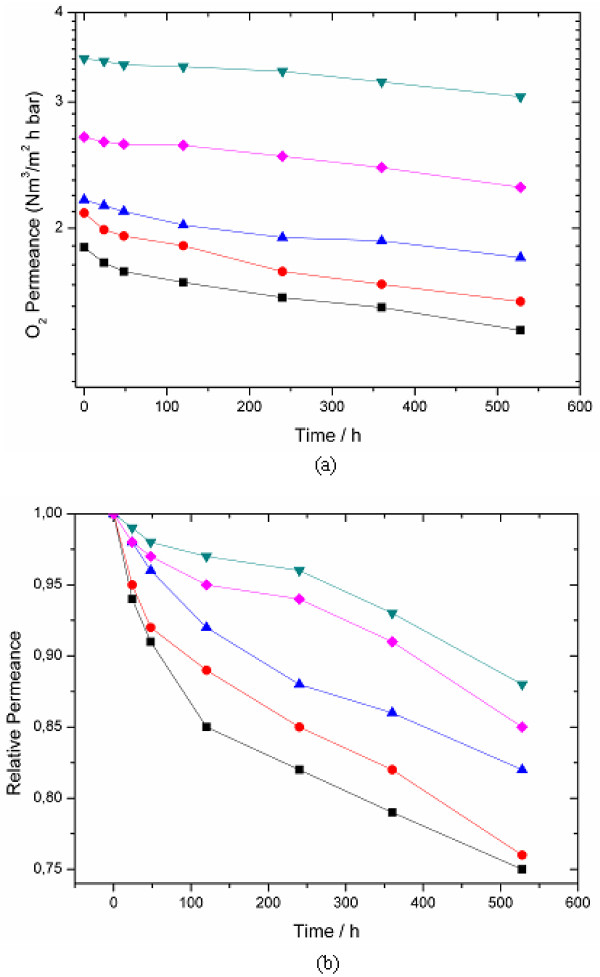
**Long term stability of PIM-1.** PIM-1 MMM incorporated with f-MWCNTs measured as (**a**) O_2_ permeance; (**b**) relative permeance. Black squares, PIM-1; red circles, 0.5 wt.% MMM; blue triangles, 1 wt.% MMM; green inverted triangles, 2 wt.% MMM; and pink diamonds, 3 wt.% MMM. Temperature, 27°C and feed pressure, 2 bar.

The long-term stability of f-MWCNTs containing MMM continuously exposed to air at 2 bar pressure was monitored and the results are shown in Figure 8. The O_2_ permeance of the fabricated membranes was measured over a period of 550 h Figure 8a. It was observed that the O_2_ permeance of f-MWCNTs MMM slowly and gradually decreased with time compared with the much more significant O_2_ permeance loss as a function of time observed for the pure PIM-1 composite membrane. The permeance trends for f-MWCNTs MMM are PIM-1 < 0.5 < 1 < 3 < 2 wt.%. Among these 2 wt.% f-MWCNTs MMM shows the better performance in long-term stability. A similar trend was found for the change of N_2_ permeance as a function of time for different f-MWCNTs MMM. Compared to PIM-1 composite membrane, the f-MWCNTs MMM tend to have better long-term stability, although none of the studied membranes has shown permeance stabilization after 550 h of continuous experiments.

Furthermore, Figure 8b shows the relative permeance data for f-MWCNTs containing MMM, where the maximum total O_2_ permeance loss was found for the pure PIM-1 composite membrane studied. The trend of 0.5 wt.% of f-MWCNTs MMM is less significant, while for the 1 and 2 wt.% f-MWCNTs MMM, the loss of permeance is very limited. From these experimental results, it can be concluded that the addition of f-MWCNTs into the polymer matrix improved the long-term stability of PIM-1.

## Conclusions

Mixed matrix membranes or MMM comprising MWCNTs as dispersed phase and PIM-1 as polymer matrix were successfully prepared. FTIR and TGA analysis have confirmed the successful PEG functionalization of MWCNTs. The SEM images of the prepared MMM revealed that the functionalized MWCNTs are well dispersed throughout the PIM-1 matrix compared to the one which is fabricated from pristine MWCNTs. It was shown that the improvement in homogeneous dispersion of MWCNTs in the MMM is due to covalent functionalization of MWCNTs with poly(ethylene glycol). With good interfacial adhesion and the absence of voids between f-MWCNTs and polymer matrix, the MMM show higher permeabilities which are coupled with increased CO_2_/N_2_ and O_2_/N_2_ selectivities. For MMM with 0.5 to 2 wt.% f-MWCNTs loading, the SEM of surface and cross-sectional images showed well dispersed f-MWCNTs throughout the polymer matrix, while at 3 wt.% of f-MWCNTs loading, the tubes agglomerated and formed domains or interface voids in the polymer matrix. Above a threshold of 2 wt.%, agglomeration and saturation of f-MWCNTs particle in the polymer matrix hinder the fast transport of gases. In addition, PEG functional groups on the surface of MWCNTs provide higher solubilities of CO_2_ in the MMM. The improvement in long-term stability was obtained as well.

## Competing interests

The authors declare that they have no competing interests.

## Authors’ contributions

MMK carried out the experiments and drafted the manuscript, GB, SS, MR contributed in useful discussions and manuscript preparation; VF and VA supervised the study. All the authors read and approved the final manuscript.
