# Erratum to: Functionalized carbon nanotubes mixed matrix membranes of polymers of intrinsic microporosity for gas separation

**DOI:** 10.1186/1556-276X-9-698

**Published:** 2014-12-29

**Authors:** Muntazim Munir Khan, Volkan Filiz, Gisela Bengtson, Sergey Shishatskiy, Mushfequr Rahman, Volker Abetz

**Affiliations:** Institute of Polymer Research, Helmholtz-Zentrum Geesthacht, Max-Planck-Strasse 1, Geesthacht, 21502 Germany

## Correction

The authors regret to have overlooked an error in (Figure five) of the original version of this article [1]. The original, incorrect form (Figure 1), and the corrected form (Figure 2) are shown in this article.

**Figure 1:**
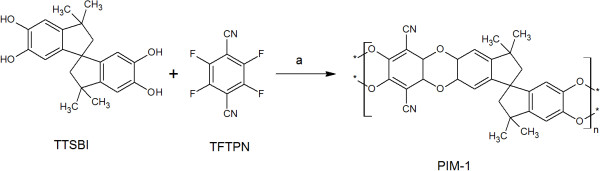
**Synthesis of PIM-1, a-reagent and condition, K2CO3, DMAc and DEB at 150°C for 1 h (Incorrect form).**

**Figure 2:**
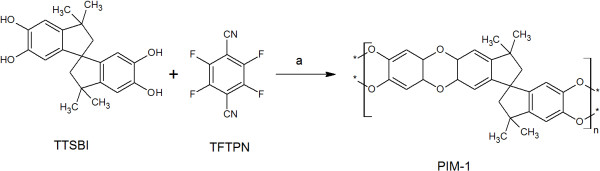
**Synthesis of PIM-1, a-reagent and condition, K2CO3, DMAc and DEB at 150°C for 1 h (Corrected form).**
